# Pleural Effusions in Pulmonary Emboli: A Single Centre Experience

**DOI:** 10.7759/cureus.11942

**Published:** 2020-12-06

**Authors:** Karl Jackson, Avinash Aujayeb

**Affiliations:** 1 Respiratory Medicine, Northumbria HealthCare NHS Foundation Trust, Newcastle, GBR

**Keywords:** pleural effusion, pulmonary embolus

## Abstract

Objectives: Pleural effusions commonly occur in conjunction with acute pulmonary emboli (PE). There are no guidelines on the investigation and management of these effusions. We aimed to review local management to inform future practice

Material and Methods: This was a retrospective, observational single centre study, looking at all computed tomography pulmonary angiograms done in a large hospital in the North East of England in 2019. Electronic notes, imaging and discharge letters of patients with PE were reviewed. Statistical analysis was performed to describe patient-specific variables, clinical characteristics, pathological findings and subsequent management decisions.

Results: 1630 computed tomography pulmonary angiographies (CTPAs) were requested to investigate for PE. Three hundred sixteen (19.4%) were positive for PE. Of those, 89 (28.1%) were identified as having associated pleural effusions. Five (5.8%) patients had a contemporaneous pleural aspiration. All pleural effusions were exudative. Four were consistent with known malignant diagnoses. The other patient had concurrent pneumonia and pancreatitis. Nineteen (21%) had no risk factors for effusion development. The presence of pulmonary infarction/consolidation was associated with the development of a pleural effusion.

Conclusions: This project is a single centre review with the largest number of patients looking at pleural effusions associated with pulmonary emboli. Although pleural effusions commonly occur with PE and lung infarction, pleural aspiration is rarely performed. Management is not altered by the presence of an effusion.

## Introduction

Pulmonary embolism (PE) has an incidence of 39-115 per 100,000 [1] with an estimated annual mortality rate of 8.3 per 100,000 [2]. PE is thought to account for over 27, 000 hospital admissions in the United Kingdom (UK) in 2011 accruing approximately 250, 000 bed days [3].

The last few years have seen a significant drive to manage conditions in an outpatient setting that were traditionally treated as inpatient. The introduction of ambulatory care units and the concept of Same Day Emergency Care (SDEC) in the UK has allowed the outpatient management of many conditions such as PE and has enabled, diagnosis, monitoring, and initiation of treatment over hours rather than days. This strategy was mentioned in the 2018 British Thoracic Society (BTS) guidelines [4].

Ambulatory management pathways are strengthened by the use of risk scores such as the Pulmonary Embolism Severity Index (PESI) score [5]. Stevenson at al. recently published a clear review of pulmonary embolism for the acute medicine department, and although the review was specifically directed toward risk stratification in the coronavirus disease 2019 (COVID-19) pandemic, it highlights the fact that PE is increasingly predominantly managed by acute medicine physicians [6]. A recent statement on the Society of Acute Medicine website is testament to this as well [7]. 

Pleural effusions have been described in approximately 19% to 61% of patients with pulmonary emboli (PE). The prevalence rate is higher if computed tomography pulmonary angiography (CTPA) is used. Current literature suggests that pleural effusions due to pulmonary emboli are associated with pulmonary infarcts, last no more than 30 days, are all exudates, and that 90% are smaller than a third of the hemi-thorax [8].

## Materials and methods

Local (Caldicott) ethical approval was granted for a single centre retrospective study in Northumbria HealthCare NHS Foundation Trust (approval C3455). All CTPAs performed in the trust between January 1 and December 31, 2019 were reviewed. Characteristics collected include age, sex, location of PE, whether an effusion was present, laterality, results of pleural taps, and subsequent management. Descriptive statistical methodology was applied to the data.

## Results

1630 CTPAs were identified. Three hundred sixteen (19.4%) were positive for PE. Of those, 89 (28.1%) had associated pleural effusions. 67 (75%) patients were managed in ambulatory care by acute medicine, and their mean S-PESI score was 2.6. Median age of the patients with pleural effusions and PE was 68 years (range 22-94, interquartile range (IQR) 19.5). There were 39 female and 60 male patients.

Pleural effusion on chest imaging

Effusions were seen on chest X-ray (CXR) in 67 patients and in CT in 80 patients, giving a sensitivity of 89.9% with CT scanning. Only the CTPAs were then analysed. Twenty-one effusions were left-sided, 24 right-sided and 44 bilateral. In the bilateral effusion group, the right side was larger than the left in two cases, and the left side larger in four instances. There were no loculations. Five effusions were large (described as occupying more than a third of the hemi-thorax).

Pleural fluid investigation

The five patients with large effusions had aspirations. All were bloodstained and exudative on analysis. Two were due to known diagnoses of lymphoma and two were due to known lung cancer diagnoses. One of the patients had bilateral effusions due to a critical illness and pancreatitis and the effusions were attributed to those.

Previous diagnoses and co-morbidities (chronic kidney disease, active cancer, critical illness, congestive cardiac failure, and hypoalbuminemia) commonly causing pleural effusions were identified. Table 1 below describes this. Some patients had more than one co-morbidity and thus the total tally might seem larger than expected but is not. For example, all the patients with critical illness had a low albumin, and 10 patients with cardiac failure also had chronic kidney disease. The patients with pancreatitis and trauma were both on critical care and had low albumin levels. Seventeen of the active cancer patients had chronic kidney disease. 

**Table 1:** Description of pleural effusions and comorbidities of patients

Effusion	Chronic Kidney disease	Active cancer	Critical illness	Congestive cardiac failure	Low albumin	Other
Unilateral (n)	7	17	5	9	8	Trauma (1)
Bilateral (n)	5	7	4	22	20	Pancreatitis (1)
No risk factors for effusion development Unilateral patients: n=16 Bilateral patients: n= 3						

Hence, 19 effusions (21%) were *presumed* to be due to pulmonary emboli but the effusions were small and not investigated further. Thirteen of those had resolved at three months, all in the unilateral group and the rest did not have repeat imaging to confirm resolution.

Sidedness of PE and pleural effusion

There was no relationship between the sidedness of the PE and the pleural effusions. The PE was unilateral in 47 patients, and the effusion ipsilateral in 21 patients, contralateral in six and bilateral in 20 patients. The PE was bilateral in 42 patients, and the effusion bilateral in 25 patients, but unilateral in the rest. Of the 16 patients who had a unilateral effusion as described in Table 1, 12 of the emboli were bilateral and four ipsilateral.

Pulmonary infarction/consolidation

Fifty-four patients had consolidation and/or infarct mentioned in the report (60.7%). Fifteen patients had no parenchymal abnormalities, 10 had atelectasis, nine ground glass changes, and one lobar collapse. Of those 19 effusions which could not be attributed to any other cause, 17 (89%) had presence of consolidation or pulmonary infarction.

## Discussion

The results of this single centre study is in line with previously known evidence. Our local CTPA positivity approaches 20% which is line with national data and the Royal College of Radiologist (UK) suggest 15.4-37.4% as an acceptable CTPA positive rate [9]. 

Porcel et al. retrospectively analyzed 230 consecutive patients with pulmonary embolism over eight years [8]. Pleural effusions were observed in 32% and 47% of patients by CXR and CT, respectively. Effusions were small and unsuitable for diagnostic thoracentesis. When sampled, all the pleural effusions due to the emboli were exudates. Peripheral wedge-shaped opacities, probably representing pulmonary infarction, were seen in 50 (38%) patients. However, pleural infarction was not associated with increased incidence of pleural effusion (50% vs. 46%, P = 0.721). There was no relationship between the side of the embolus and the effusion. There is no discussion on the associated co-morbidities that might lead to pleural effusions.

Panjwani et al. screened 1756 CTPAs over a three-year period with a diagnosis of acute PE in 200 patients (11.4%) [10]. Pleural effusions were identified in 70 (35%). These were small to moderate in size, bilateral, and associated with peripheral emboli. Consolidation, atelectasis, and ground glass attenuation were the most associated findings. However, consolidation was more common in patients with pulmonary emboli and pleural effusions as compared to those with pulmonary emboli alone. Diagnostic thoracentesis was done in six (8.6%) and all the effusions were exudative.

Lui et al. identified 423 (13.5%) patients with pleural effusions and pulmonary emboli after screening 3141 patients with clinically suspected pulmonary embolism who underwent CTPA [11]. Pleural effusions were commoner in patients with pulmonary embolism (19.9%) than without (9.4%, P < 0.001). Most of the pleural effusions in pulmonary embolism were small to moderate and unilateral. Location of emboli, arteries involved, CT pulmonary obstruction index, and parenchymal abnormalities were not associated with pleural effusions.

Previous reviews of the literature suggest that these effusions can be unilateral despite bilateral embolic disease or bilateral when the pulmonary embolism is unilateral [12-14]. The effusion can also be unilateral and contralateral in approximately 7.5% of patients [12]. Pleural effusions due to pulmonary emboli are exudates, neutrophilic, and blood stained [13,14]. Postulated mechanisms are that the emboli increase right ventricular pressure leading to an increase of the systemic venous pressure (causing pulmonary hypertension) at the parietal pleural surface which in turn increases permeability. It is also thought that the occlusion of the artery by the emboli leads to distal ischemia and cytokine release which in turn leads to vessel permeability and to an increase of interstitial fluid in the lung which can spill over into the pleural space [14].

Our study used CT to determine the presence of a pleural effusion rather than chest radiographs which greatly increases the pick-up rate, and we have a relatively large sample size. We were able to go back to the medical records and note if any other associated conditions or co-morbidities could cause the pleural effusions. The data were double-checked by the pleural lead of the local trust. Common causes of pleural effusion such as chronic kidney disease, congestive cardiac failure, critical illness, low albumin states, and active cancer states were identified and associated in patients with unilateral as well as bilateral pleural effusions. Nineteen patients with emboli and pleural effusions had no attributable cause, and perhaps in our study, the true incidence of pleural effusions due to pulmonary emboli is 21%. However, none of those were investigated further and this is the main limitation of this study which has been previously described. The majority of effusions secondary to pulmonary embolism are small, which preclude a diagnostic and therapeutic thoracentesis. Many patients on the clinical triage pathways are anticoagulated while awaiting a confirmatory test and the effusions gradually resolve, rendering a thoracentesis unnecessary. A prospective study by Hooper et al. found an incidence of 6.4% of pulmonary embolism in patients with unilateral pleural effusion indicating that pulmonary embolism is not a common primary cause for unilateral pleural effusion [15]. Their findings are corroborated with ours that when present, pleural effusion is frequently associated with pleural malignancy and malignancy-related effusions.

The 19 patients with pleural effusions that could not be attributed to any other cause other than pulmonary emboli had pulmonary infarcts or consolidation on CT scanning. A pulmonary embolus will cause diminished blood flow to the distal lung parenchyma, causing ischemia which in turn leads to infarction and necrosis.

The main limitation of our study is that we did not compare findings of patients with emboli and pleural effusions to patients with pulmonary emboli and without pleural effusions and this statistical analysis is limited to basic descriptive methodology.

## Conclusions

Our retrospective analysis is one of the largest in the literature and adds to the evidence base that pleural effusions are often present in the context of pulmonary emboli, that the effusions are mostly small and can often be explained by other concurrent co-morbidities. There was no relationship to the side of the embolus and effusion. Pulmonary infarction/consolidation appears to be more common with pulmonary emboli and pleural effusions. Management is not usually altered by the presence of an effusion with a pulmonary embolus.
